# Immunological barriers to immunotherapy in primary and metastatic breast cancer

**DOI:** 10.15252/emmm.202114393

**Published:** 2021-06-15

**Authors:** Mara De Martino, Claire Vanpouille‐Box, Lorenzo Galluzzi

**Affiliations:** ^1^ Department of Radiation Oncology Weill Cornell Medical College New York NY USA; ^2^ Sandra and Edward Meyer Cancer Center New York NY USA; ^3^ Caryl and Israel Englander Institute for Precision Medicine New York NY USA; ^4^ Department of Dermatology Yale School of Medicine New Haven CT USA; ^5^ Université de Paris Paris France

**Keywords:** CTLA4, glioblastoma, MPA/DMBA‐driven carcinomas, PD‐1, TGF‐β, Cancer, Immunology

## Abstract

Patients with breast cancer obtain limited clinical benefits from immune checkpoint inhibitors (ICIs), pointing to the existence of multiple immunological alterations that cannot be simultaneously normalized with immunotherapy. Accumulating preclinical evidence suggests that radiation therapy (RT) can be harnessed to sensitize primary and metastatic mouse mammary carcinomas to ICIs. However, various clinical trials combining RT with ICIs in patients with breast cancer documented little cooperativity. Here, we discuss immunological barriers that may prevent RT from unlocking the therapeutic potential of ICIs in patients with breast cancer. These observations may inspire the development of combinatorial regimens that might benefit patients with diverse neoplastic conditions including brain tumors.

## Graphical abstract

In this Commentary, L. Galluzzi and colleagues discuss the immunological barriers that may prevent radiotherapy from unlocking the therapeutic potential of immune checkpoint inhibitors in patients with breast cancer.

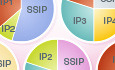

Despite considerable expectations driven by the clinical success of immune checkpoint inhibitors (ICIs) in patients with various solid tumors (*e.g*., melanoma, lung carcinoma), women with breast cancer (BC) obtain limited benefits from ICI‐based immunotherapy (Emens, 2018). The realization that ICIs employed as stand‐alone immunotherapeutic agents are virtually ineffective in patients with BC has spun a considerable experimental effort aimed at the identification of combinatorial regiments that would unlock the therapeutic potential of ICIs. In multiple preclinical models of primary and metastatic BC, radiation therapy (RT) emerged as a promising combinatorial partner for ICI‐based immunotherapy (De Martino *et al*, 2021), driving the initiation of various clinical trials investigating RT plus ICIs in women with advanced or metastatic BC. Unfortunately, most of these studies document little, if any, advantage from combining RT with ICIs in patients with BC, even in the triple‐negative BC (TNBC) setting, in which the abundance of tumor‐infiltrating lymphocytes (TILs) has a major prognostic value (Voorwerk *et al*, 2019). Thus, pre‐existing or newly emerging immunological mechanisms must be at play in the microenvironment of primary and metastatic BC lesions to prevent the efficacy of ICIs employed alone or combined with RT. Here, we briefly discuss preclinical data identifying barriers that may impede the immunological eradication of BC as well as other immunotherapy‐resistant tumors, such as brain neoplasms.

Multiple preclinical models of BC that can be harnessed for immuno‐oncology and immunotherapy studies, including mouse mammary carcinoma TS/A (a common model of luminal BC) and 4T1 (a common model of TNBC) cells established subcutaneously, as well as endogenous mammary carcinomas driven in immunocompetent mice by medroxyprogesterone acetate (MPA, M) and 7,12‐dimethylbenz[a]anthracene (DMBA, D) or by the middle T polyoma antigen (PyMT) expressed under the control of the MMTV promoter (two additional models of luminal BC), recapitulate the intrinsic insensitivity of their human counterparts to ICIs targeting cytotoxic T lymphocyte‐associated protein 4 (CTLA4) or programmed cell death 1 (PDCD1, best known as PD‐1; Buque *et al*, 2020; Yamazaki *et al*, 2020; De Martino *et al*, 2021; Niesel *et al*, 2021). In most of these models, combining RT with CTLA4 or PD‐1 blockers considerably extends the therapeutic benefits of RT employed as stand‐alone intervention, especially with respect to the control of systemic (non‐irradiated) lesions, or brain metastases receiving otherwise ineffective whole‐brain RT (WBRT), ultimately translating into a survival benefit (Yamazaki *et al*, 2020; De Martino *et al*, 2021; Niesel *et al*, 2021). Nonetheless, virtually all mice bearing 4T1, M/D‐driven, or MMTV‐PyMT derived (99LN cells) mammary carcinomas and treated with RT plus ICIs ultimately succumb to the disease (Buque *et al*, 2020; De Martino *et al*, 2021; Niesel *et al*, 2021). Conversely, a fraction of mice bearing two *s.c*. TS/A lesions (to model oligometastatic disease) experience systemic disease eradication upon focal RT to one lesion plus CTLA4 or PD‐1 blockage (De Martino *et al*, 2021), despite the fact that TS/A cells are generally viewed as poorly immunogenic. These observations suggest that the skin may be more permissive for the development of robust BC‐targeting immune responses as compared to the lungs (the preferential site of dissemination for 4T1 cells) or the brain (the preferential site of dissemination for intracardially administered 99LN cells). Interestingly, 4T1 cells as well as 67NR cells (a model of luminal BC) established orthotopically in the mammary fat pad appear to respond, at least to some degree, to ICIs targeting PD‐1 and its main ligand CD274 (PD‐L1; Hubert *et al*, 2021). Although this feature is not shared with human BC, and allografts do not properly recapitulate oncogenesis and tumor progression in the context of failing immunosurveillance, the mouse mammary microenvironment may represent a privileged source of information to elucidate immunological mechanisms that enable ICI efficacy.

That said, it seems that both endogenous mouse mammary carcinomas (which develop orthotopically by definition) and metastatic allografts preserve the ability to evade tumor‐targeting immunity driven by RT in combination with ICIs, most likely as a consequence of local immunosuppression. Abundant preclinical literature indicates that this capacity reflects not only pre‐existing features of the disease, but also immunosuppressive pathways elicited by treatment. For instance, optimal anticancer immunity driven by RT requires proficient type I interferon (IFN) signaling as a consequence of cytosolic double‐stranded DNA (dsDNA) accumulation in irradiated malignant cells, and (at least in preclinical settings) this is actively counteracted by autophagy, which operates at baseline in all cells but is upregulated by RT (Yamazaki *et al*, 2020), as well as by three prime repair exonuclease 1 (TREX1), an exonuclease that (in most cell types) is elicited at RT doses > 10–12 Gy (De Martino *et al*, 2021). Along similar lines, the ability of RT to synergize with ICIs at the initiation of robust tumor‐targeting immune responses against experimental BC is inhibited by transforming growth factor beta (TGF‐β), which is released from the tumor stroma as an active molecule upon RT, and inhibin subunit beta A (INHBA), which recruits immunosuppressive cells including (but not limited to) CD4^+^CD25^+^FOXP3^+^ regulatory T (T_REG_) cells (De Martino *et al*, 2021) and bone marrow‐derived PD‐L1‐expressing myeloid cells (Niesel *et al*, 2021). Of note, some of these detrimental effects of RT alone (such as the recruitment of T_REG_ cells) are actively counteracted by ICIs, while others (such as the recruitment of PD‐L1 expressing myeloid cells) are aggravated by them (Niesel *et al*, 2021), highlighting potential targets for the development of combinatorial regimens with superior activity in patients. However, the immunosuppressive circuitries established by primary and metastatic BCs appear to be highly multilayered in nature, implying that “simple” combinatorial regimens may not be sufficient to reconfigure the immune contexture of the tumor microenvironment in support of robust anticancer immunity. In line with this notion, combining RT with a TGF‐β‐targeting antibody (*i.e*., fresolimumab) in patients with metastatic BC was associated with systemic signs of tumor‐targeting immunity and a trend toward improved overall survival (despite a limited objective response rate) only in individuals receiving fresolimumab at 10 mg/Kg (Formenti *et al*, 2018). Moreover, anatomical localization appears to influence considerably the immunological configuration of progressing tumors, suggesting that site‐tailored interventions may be necessary to maximize efficacy.

The brain is among the most common sites of metastatic dissemination in patients with BC, and brain metastases are a frequent cause of BC‐related deaths. Although the brain has long been viewed as an immunologically privileged site with little infiltration by circulating immune cells, it is now clear that immunosuppressive circuitries established by primary brain tumors, notably gliomas and glioblastomas (GBMs), as well as by brain metastases from extracranial malignancies are major driver of disease progression and resistance to ICI‐based immunotherapy (Lopez Vazquez *et al*, 2021). Patients with brain tumors frequently receive RT as part of disease management, but aggressive brain neoplasms such as high‐grade GBM respond poorly to RT. Preclinical data indicate that targeting PD‐L1‐expressing myeloid cells synergizes with RT in the control of experimental syngeneic glioblastomas (Zhang *et al*, 2019), drawing an interesting parallel with brain metastases from BC (Niesel *et al*, 2021).

Indeed, GBM and BC‐derived brain lesions share the ability to recruit immunosuppressive myeloid cells expressing colony‐stimulating factor 1 receptor (CSF1R), ultimately generating a population of tumor‐associated macrophages (TAMs) that are dynamically altered during therapy. In this context, RT has been shown to promote recurrence‐specific phenotypes in microglial cells and monocyte‐derived macrophages (MDMs) that support GBM proliferation and disease relapse (Akkari *et al*, 2020). These data identify the need to account for the plasticity and flexibility of CSF1R^+^ myeloid cells to maximize the efficacy of therapeutic agents targeting neoplastic lesions in the brain. Supporting this concept, a CSF1R‐targeting antibody considerably enhances the efficacy of focal RT given in five daily fractions of 2 Gy each in preclinical GBM models, further extending the survival of GBM‐bearing mice (Akkari *et al*, 2020). While CSF1R‐targeting strategies are currently being evaluated in patients with GBM (NCT02829723), CSF1R inhibition has also shown some promise in patients with advanced refractory BC bearing brain metastases (Autio *et al*, 2020), further underscoring the similarities between GBM and BC‐derived brain lesions.

Yet another barrier to the efficacy of RT and immunotherapy in patients with brain neoplasms is the limited infiltration of malignant lesions by T cells, largely reflecting (i) thymic involution, (ii) bone marrow sequestration, (iii) increased expression of PD‐L1 by cancer cells, and (iv) the loss of MHC Class II expression by CD68^+^ microglial cells. Of note, a large proportion of T cells that successfully infiltrate intracranial tumors is represented by T_REG_ cells co‐expressing CTLA4 and PD‐1. As the activation of TGF‐β signaling that accompanies gliomagenesis (yet another GBM feature shared with BC) might account for this observation, TGF‐β‐targeting strategies may also need to be incorporated in combinatorial regimens to unlock the efficacy of RT and ICIs in patients with GBM. Indeed, TGF‐β blockage alone proved inefficient in clinical trials enrolling patients with GBM, in thus far resembling ICI‐based immunotherapy.

Taken together, these observations delineate a multilayered panel of barriers that must be overcome to unleash the full therapeutic potential of ICIs in patients with primary or metastatic BC and other tumors that are poorly sensitive to immunotherapy, such as brain cancer (Fig 1). We surmise that anatomical disease localization plays a major and hitherto underappreciated role in the establishment of such barriers and hence will have to be taken under attentive consideration for the development of combinatorial regimens with superior efficacy.

**Figure 1:**
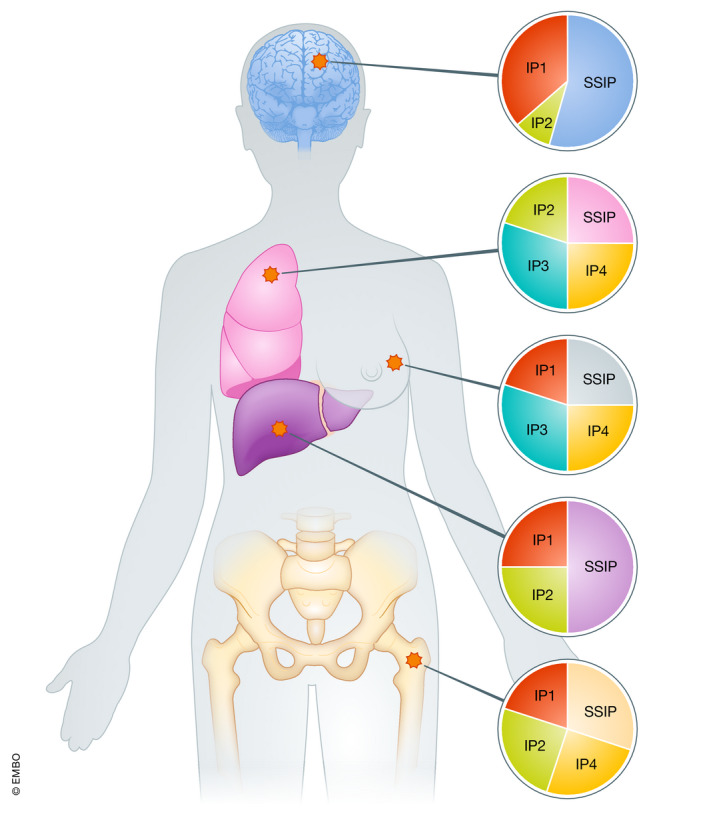
Impact of anatomical site on immunotherapy resistance Primary and metastatic breast tumors establish a number of immunosuppressive circuitries in support of disease progression and resistance to immunotherapy. Such immunosuppressive pathways (IP1, IP2, etc.) are generally multilayered in nature and differ between primary and metastatic disease sites, which considerably complicates the development of combinatorial therapeutic regimens that unlock the efficacy of immunotherapy. Interestingly, it seems that tumors of different histology developing at the same site rely on relatively similar immunosuppressive mechanisms for progressing and resisting treatment, pointing to a major role for anatomical location in the establishment of local immunosuppression. Please note that the relative contribution of IPs and site‐specific IPs (SSIPs) depicted here is for exemplifying purposes and does not reflect existing preclinical or clinical data.

## Conflict of interest

CV‐B and MDM have no conflicts of interest to declare. LG reports research funding from Lytix, and Phosplatin (completed), and consulting/advisory honoraria from Boehringer Ingelheim, AstraZeneca, OmniSeq, The Longevity Labs, Inzen, and the Luke Heller TECPR2 Foundation.
